# Recent Clinical Advances on Long Non-Coding RNAs in Triple-Negative Breast Cancer

**DOI:** 10.3390/cells12040674

**Published:** 2023-02-20

**Authors:** Desh Deepak Singh, Hae-Jeung Lee, Dharmendra Kumar Yadav

**Affiliations:** 1 Amity Institute of Biotechnology, Amity University Rajasthan, Jaipur 303002, India; 2 Department of Food and Nutrition, College of Bionano Technology, Gachon University, Seongnam-si 13120, Republic of Korea; 3 Arontier Co., Seoul 06735, Republic of Korea

**Keywords:** triple-negative breast cancer, lncRNA, diagnosis, targeted drug development and resistance

## Abstract

Triple-negative breast cancer (TNBC) is a more aggressive type of breast cancer due to its heterogeneity and complex molecular mechanisms. TNBC has a high risk for metastasis, and it is difficult to manage clinical conditions of the patients. Various investigations are being conducted to overcome these challenges using RNA, DNA, and proteins for early diagnosis and treatment. Recently, long non-coding RNAs (lncRNAs) have emerged as a novel target to treat the multistep process of TNBC. LncRNAs regulate epigenetic expression levels, cell proliferation and apoptosis, and tumour invasiveness and metastasis. Thus, lncRNA-based early diagnosis and treatment options could be helpful, especially for patients with severe TNBC. lncRNAs are expressed in a highly specific manner in cells and tissues and are involved in TNBC progression and development. lncRNAs could be used as sensitive and specific targets for diagnosis, treatment, and monitoring of patients with TNBC. Therefore, the exploration of novel diagnostic and prognostic biomarkers is of extreme importance. Here, we discuss the molecular advances on lncRNA regulation of TNBC and lncRNA-based early diagnosis, treatment, and drug resistance.

## 1. Introduction

**Figure 1:**
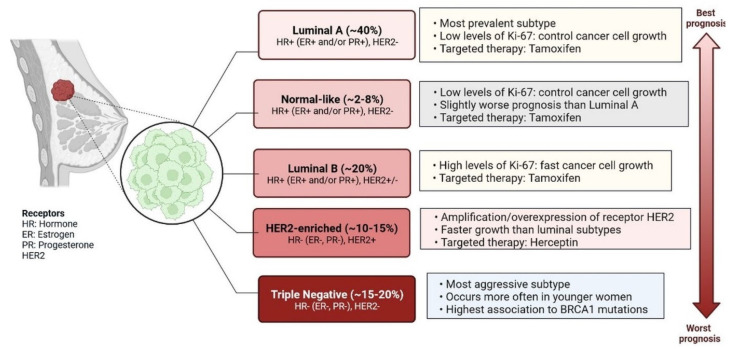
Main intrinsic or molecular subtypes of breast cancer. There are five main intrinsic or molecular subtypes of BC such as Luminal A (~40%) [HR+ (ER+ and/or PR+), HER2-], Normal-like (~2–8%) [HR+ (ER+ and/or PR+), HER2-], Luminal B (~20%) [HR+ (ER+ and/or PR+), HER2+/−], HER2-enriched (~10–15%) [HR− (ER−, PR−), HER2+], and Triple-negative (~15–20%) [HR- (ER−, PR−) HER2].

**Figure 2:**
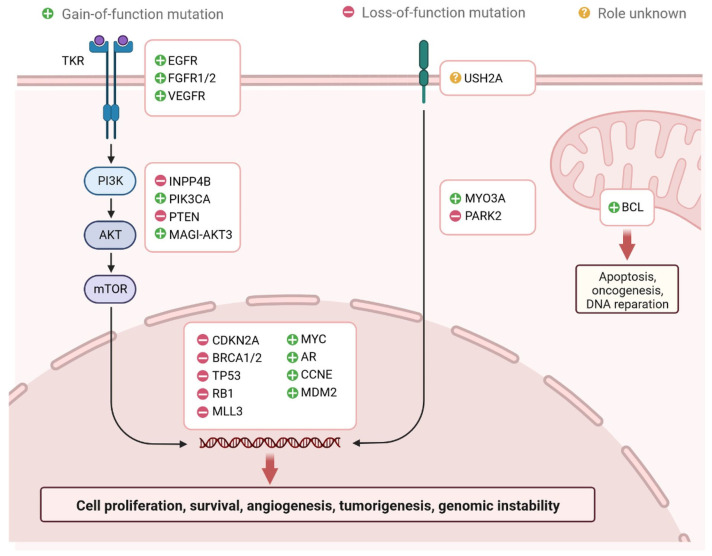
Regulation of genetic markers associated with TNBC. Genetic markers associated with gain of function: EGFR, EGFR1/2, VEGFR, MYC, AR, CCNE, MDM2, PIK3CA, MAGI-AKT3, MYO3A, and PARK2. Genetic markers associated with Loss of function: INPP4B, PTEN, CDKN2A, BRCA1/2, TP53, RB1, and MLL3.

Breast cancer (BC) is caused by epigenetic modifications and is a highly heterogeneous disease. It exhibits various histological and clinical features [1]. There are five main intrinsic molecular subtypes of BC. To my knowledge, the five intrinsic molecular subtypes are: Luminal A, Luminal B, HER2-enriched, Triple-negative/Basal- like, and Claudin-low. Among these, Luminal A breast cancers are HER2-negative (HR+/HER2−) and include ER+/PR+, ER+/PR−, and ER−/PR+ status. (Figure 1) [2]. Triple-negative breast cancer (TNBC) is the most aggressive molecular clinical subtype of all invasive cancers. Various genetic markers are expressed during the development of TNBC (Figure 2) [3], which are involved in a gain or loss of function. Genetic markers involved in gain of function include EGFR, EGFR1/2, VEGFR, MYC, AR, CCNE, MDM2, PIK3CA, MAGI-AKT3, MYO3A, PARK2, and genetic markers involved in loss of function are INPP4B, PTEN, CDKN2A, BRCA1/2, TP53, RB1, and MLL3 (Figure 2) [4]. Advances in genetic heterogeneity research have revealed prognostic and therapeutic targets in TNBC [1,2,3]. TNBC has been divided into four distinct subtypes using gene expression analysis: basal-like immune-activated (BLIA), basal-like immune-suppressed (BLIS), mesenchymal (MES), and luminal androgen receptor (LAR). The classification of TNBC subtypes lacks a standardised system, however. Basal-like (BL) tumours make up around 80% of TNBC tumours, and because BL tumours cluster physiologically apart from the other BC subtypes, intrinsic subtyping is less effective for significant diagnosis and classification than it is for the other clinical subtypes. Multiple initiatives to investigate DNA, RNA, microRNA, and protein expression patterns through cross-platform research such as the Cancer Genome Atlas have provided more comprehensive evidence of BC heterogeneity. TNBC subtyping is useful for classifying patients for individualised care. However, research is ongoing, and no laboratory approach for classifying TNBC subtypes has yet been used in clinical settings. The overall malignancy indicated above was used to evaluate the initial and modified versions of the Lehman classifier, and the results indicated that non-cancer cells strongly influence the gene expression profiles that affect treatment response and prognosis in TNBC. The interrelations between cancer cells and the immune system, originally known as “immune surveillance”, provide an excellent example of the significance of each element of the tumour microenvironment on behaviour and prognosis. Not all patients respond well to immunotherapy, even though TNBC is the most immune-activated subtype of all BC, as shown by immune gene expression as well as levels of intra- and stromal tumour-infiltrating lymphocytes (TILs). The appropriate way to define immune activation is still being worked out, but current methods include looking for TILs, producing the protein programmed death ligand 1 (PDL1), immune gene signatures, individual immune gene RNA expression, and immune cell clonality (T cells and B cells) investigations. The TNBC subtype is still requires further investigations to improve treatment responder binding proteins are highly conserved, and binds with coding and non-coding RNAs through their RNA binding domain and metabolic process such as RNA splicing, polyadenylation, localization, translation, and destruction. Various types of non-coding RNAs (ncRNAs) have been investigated, including microRNAs (miRNAs), ribosomal RNAs (rRNAs), transfer RNAs (tRNAs), small interfering RNAs (siRNAs), small nuclear RNAs (snRNAs), extracellular RNAs (exRNAs), small Cajal body-specific RNAs, circular RNAs (circRNAs), and long non-coding RNAs (lncRNAs) [5,6,7,8,9,10,11,12]. ncRNAs are seen in a variety of malignancies, which are actively involved in cell proliferation and inhibition of tumour development [8]. RBPs are actively involved in regulations of TNBC at the transcriptional and post-transcriptional level [8]. The hnRNP E1 RBPs interact with PNUTS pre-RNA and suppress the splicing of lncRNA-PNUTS and regulates EMT (epithelial–mesenchymal transition) and promotes tumour development via interacting with miR-205 as a competitive sponge [8,9]. Additionally, N6-methyladenosine (m6A) readers, writers, and erasers are all RBPs that vary the functionality of lncRNAs by changing their expression levels. RBPs have different mechanisms to regulate the expression of lncRNAs. Breast cancer prognostic markers for autophagy, aerobic glycolysis, stemness, and immune-related lncRNA have been established [8,9]. Transcribed genomes can produce thousands of lncRNAs, which contain more than 200 nucleotides. The first lncRNA was identified in 1990 in a mouse model [6]. lncRNAs are found in cytosolic or nuclear regions as interspersed, overlapping regions of coding and non-coding transcripts. They have different molecular functions and roles, including molecular signalling, scaffolding, acting as decoys, integrating developmental signals, clarifying the cellular context, guiding gene expression, or responding to different stimuli [7]. Understanding of scaffolding complexes would provide novel strategies for the implementation of specific signalling components to alter molecular processes [7,8]. Sense, antisense, and bidirectional as well as intronic and intergenic lncRNAs have been observed, and they all participate in various cellular processes. lncRNAs, including NRON, HEIH, HCP5, LINC00096, growth-stasis-specific transcript 5 (GAS5), NEAT1, AWPPH, LUCAT1, HAND2-AS1, POU3F3, MALAT1, and ANRIL, are actively involved in TNBC apoptosis and proliferation [10,11,12,13,14,15,16]. All these lncRNAs could be potential targets for diagnosis and drug development against TNBC cells. Several studies have reported that lncRNAs play important roles in TNBC disease progression through various gene regulatory mechanisms and the induction of intramolecular interactions. The aberrant expression of lncRNAs is involved in TNBC initiation, progression, and metastasis, and affects various biomarkers. Therefore, lncRNAs are important for early diagnosis and the clinical management of patients.

## 2. LncRNAs

**Figure 3:**
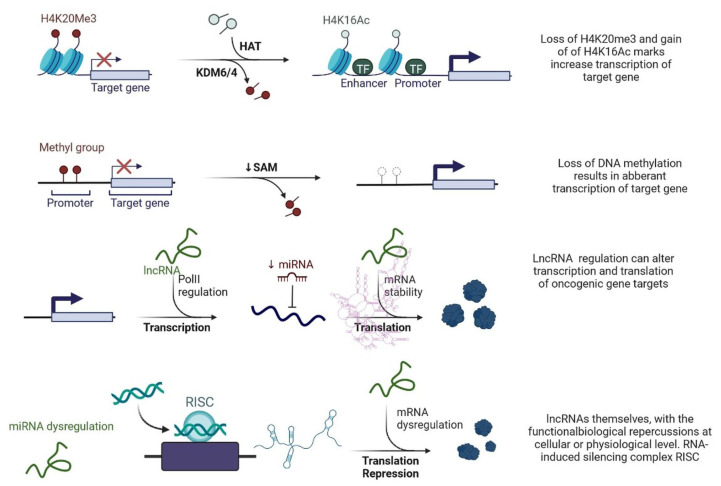
Epigenetic deregulation in cancer including chromatin remodelling, DNA methylation, and non-coding RNA regulation that alters transcription and translation of oncogenic gene targets.

**Figure 4:**
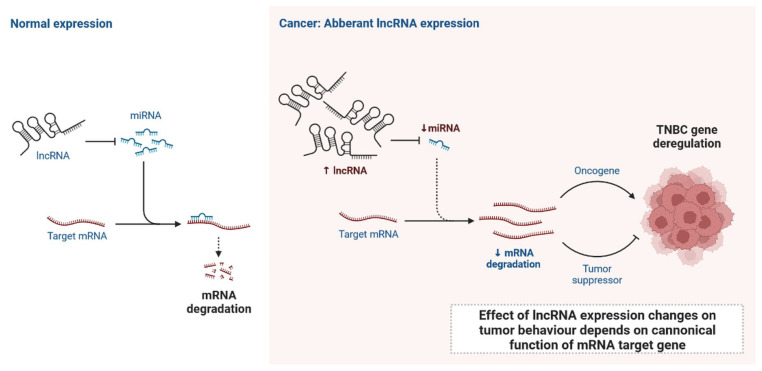
Normal expression of lncRNA and effect of lncRNA expression changes on tumour behaviour depends on canonical function of mRNA target gene.

**Figure 5:**
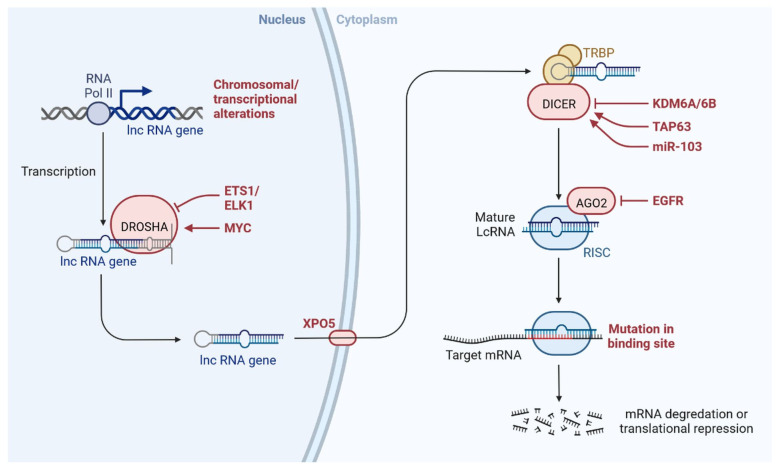
lncRNAs are involved with the functional repercussions at the cellular and physiological level. RNA-induced silencing complex (RISC): KDM5B (lysine-specific demethylase 5B also known as histone demethylase JARID1B), H3K4me3 (trimethylation of lysine 4 on the histone H3 protein subunit), H3K4me1 (monomethylation of lysine 4 on the histone H3 protein subunit), hsa-miR-448 (also known miRNA448), BRCA1/2 (breast cancer 1/2), pRB (retinoblastoma protein), CAV 1 (caveolin 1), HOXA5 (Homeobox protein Hox-A5), SFN (Stratifin), CH3 (methyl group), and RhoA (Ras homolog gene family, member A).

lncRNAs are actively involved in gene expression, epigenetic deregulation, chromatin remodelling, DNA methylation, translation of oncogenic gene targets, and biogenesis (Figure 3). They are transcribed by RNA polymerase II, after which most transcripts are spliced, and are mainly found in the nucleus and chromatin, being expressed in cells and tissues in a specific manner [6,9,17]. Transcriptional regulation and various molecular processes in the cytoplasm are controlled by lncRNAs; various circulating lncRNAs are transmitted via exosomes and bind to various transcription factors, chromatin-regulated complexes, RNA-binding proteins, nascent RNA transcripts, and chromatin [17]. The normal expression of lncRNAs and the effect of their expression changes on tumour behaviour depends on the canonical function of the mRNA target genes (Figure 4). lncRNAs can bind to the active site of proteins and regulate molecular processes at the post-transcriptional level. They are involved in functional biological processes at the cellular or physiological levels. RNA-induced silencing complexes (RISCs) are formed with the help of lysine-specific demethylase 5B (KDM5B, also known as histone demethylase JARID1B), trimethylation of lysine 4 on the histone H3 protein subunit (H3K4me3), monomethylation of lysine 4 on the histone H3 protein subunit (H3K4me1), hsa-miR-448 (also known as miRNA448), breast cancer 1/2 (BRCA1/2), retinoblastoma protein (pRB), caveolin-1 (CAV-1), Homeobox protein Hox-A5 (HOXA5), Stratifin (SFN), methyl groups (CH3), and Ras homolog gene family, member A (RhoA) (Figure 3 and Figure 5) [18]. In 2019, it was found that the lncRNA MIR100HG regulates proliferation in TNBC and the expression of the p27 gene after formation of an RNA–DNA triplex at the promoter [19]. Moreover, MIR100HG silencing leads to reduced transcription and translation of p27 [19,20]. Three triplex-forming oligonucleotides (TFOs) have been observed on the lncRNA of p27, which binds to the triplex-targeting ability (TTA) site at the 5’UTR; this event has been observed in TNBC cell lysates [21]. The binding of TFO1 and TTA is a unique mechanism by which MIR100HG regulates the transcription factors at the promoter region of p27 [21,22]. Plasmacytoma variant translocation 1 (PVT1) is another type of lncRNA that is transcribed by a gene situated at the 8q24 chromosomal region and plays and important role in TNBC development. It contains 12 exons that when spliced generate lncRNAs [23]. PVT1 binds to Krüppel-like factor 5 (KLF5) and generates a BAP1 deubiquitinase that induces TNBC via beta-catenin upregulation. Furthermore, the PVT1 promoter also acts as a regulator of the expression of the MYC proto-oncogene and BHLH transcription factor (c-MYC) [24]. These findings show that lncRNAs also mediate regulation at the transcriptional level.

## 3. Clinical Updates on lncRNAs in TNBC

**Figure 6:**
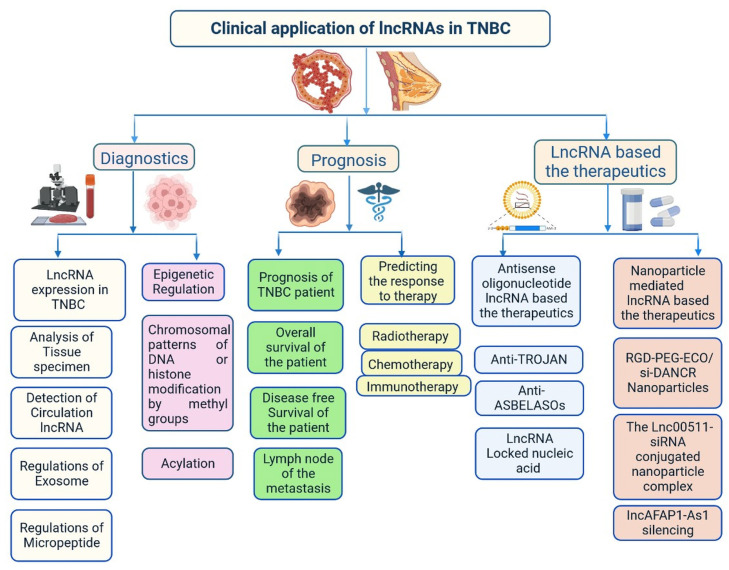
Clinical importance of lncRNA in triple-negative breast cancer.

Recently, lncRNA expression in patients with TNBC was investigated; 1034 lncRNAs were identified using NGS technologies and microarrays, out of which, 537 lncRNAs regulate 451 protein-coding genes [14]. These genes are also detected in TNBC cells and are involved in cell signalling pathways such as the MAPK and PI3K-Akt pathways, which may lead to heterogeneity [14,24]. lncRNAs also act as miRNAs, binding to miRNA-targeted mRNAs and dysregulated miRNAs [25]. This crosstalk forms a complex post-transcriptional regulatory network including mRNAs and lncRNAs that is called the competing endogenous RNA (ceRNA) network [26]. ceRNA-mediated regulatory mechanisms constitute an important pathway in lncRNA-modulated post-transcriptional regulation in TNBC [27]. A microarray-based ceRNA network analysis revealed that 4852 lncRNAs are related to the diagnosis and treatment outcome of TNBC [28]. Another study using the TCGA database found that 150 lncRNAs are expressed at the tissue level and 823 in serum and these lncRNAs could act as prognostic factors in TNBC [29]. Furthermore, the study found that the lncRNA OSTN-AS1 is a novel immune-related prognostic marker [29]. An integrated ceRNA network involving three miRNAs (CHRDL1, FCGR1A, and RSAD2) and two lncRNAs (HIF1A-AS2 and AK124454) was developed using microarray analysis [30]. These findings demonstrate that lncRNAs play major roles in the regulation of cell signalling, genetic heterogeneity, TNBC development, and pathological features (Figure 6) shown in Table 1.

### 3.1. Importance of lncRNAs in Tumour Invasiveness and Metastasis

Tumour invasion and metastasis explain the severity and mortality rate in patients with TNBC (Figure 6) [78,79]. GAS5 overexpression induces the expression of miR-196a-5p, which activates the FOXO1/PI3K/Akt signalling pathway [80]. TROJAN is a drug that reduces the metastasis burden. Degradation of TROJAN is regulated by ZMYND8, and the ubiquitin–proteasome pathway is involved in this process [81]. CCAT1 activates the migration of TNBC cells via miR-218/ZFX signalling [40]. Various ncRNAs are involved in cell migration and invasion via specific regulatory pathways, including MIR503HG through the miR-103/OLFM4 axis [60], CCAT1 through the dysregulation of the miR-218/ZFX axis [40], AFAP1-AS1 through the activation of Wnt/β-catenin signalling [82], miR-34a through the activation of EMT-associated signalling pathways [83], PAPAS through miR-34a.83 downregulation [52], sONE through sONE/NOS3/NO signalling activation [53], LINC-ZNF469-3 by activating the miR-574-5p/ZEB1 axis [71,78], ZEB2 through the activation of PI3K/Akt/GSK3β/ZEB2 signalling [45], PVT1 by regulating p21 and KLF5/β-catenin signalling [24], ARNILA by mimicking ceRNA for miR-204, AIRN by downregulating Wnt/β-catenin/mTOR/PI3K signalling [36], RMST by downregulating Wnt/β-catenin/mTOR/PI3K signalling [67], and MALAT1 by upregulating miR-129-5p and miR-1/Slug expression [84]. Furthermore, miR-448 and some other lncRNAs play very important roles in invasion and metastasis, including SKAI1BC, HULC, HOTAIR, SNHG12, SNAR, WT1-AS, LINC01096, DANCR, NEF, HIF1A-AS2, LncKLHDC7B, and ROR [30,31,32,38,48,55,58,59,60,61,62,63,64,65,66,67,68,69,85,86].

### 3.2. Importance of lncRNAs in Clinical Diagnosis

Several studies have found that lncRNAs are involved in the regulation of various transcription factors, epigenetic changes, chromatin remodelling, DNA methylation patterns, alternative splicing, post-translational modifications, and interaction with small peptides. All these events have great importance in the early diagnosis and treatment of patients with TNBC [14,86]. lncRNA expression levels in the blood and tissues of patients with TNBC at different stages has been investigated [14]. Based on reverse transcription quantitative PCR analysis data, the lncRNAs HIF1A-AS2, UCA1, and ANRIL can be used for TNBC detection, with areas under the curve in the range of 0.827–0.840, and a diagnostic accuracy of 0.962 for ANRIL [87]. ANRIL, SOX2OT, and ANRASSF1 are used to differentiate between healthy and TNBC cells. TINCR expression is used to differentiate various histological subtypes of BC, as it is highly expressed in TNBC cells [88]. UCA1 is associated with TNBC, acting as a specific marker for TNBC diagnosis. EZH2 is highly expressed in TNBC tissues and prevents apoptosis by activating the miR-4458/SOCS1 axis [89]. LINC00299 expression is increased in TNBC. Several lncRNAs bind to mRNAs, protecting them and increasing their stability. The oncogenic transcription factor SOX9 is activated by LINC02095 [90]. DANCR interacts with RXRA and activates PI3K/Akt signalling in TNBC [58]. LINC00152 enhances NEDD4-1-facilitated ubiquitination and dysregulation of PTEN protein in TNBC [91]. Cell cycle arrest at the G1 phase is induced by MIR100HG, with p27 binding to RNA–DNA; p27 is a cyclin-dependent kinase (CDK) inhibitor. Cell cycle arrest at the G0/G1 phase is induced by LINC00339 and RMST in TNBC through the miR-377-3p/HOXC6 signalling pathway [19,20,77,92]. GAS5 is actively involved in the inhibition of TNBC cells through its action on miR-196a-5p and miR-378a-5p/SUFU signalling [93]. Further understanding of the roles of all these lncRNAs in TNBC is needed to improve early diagnosis and clinical management of patients. Various genes are targeted by ncRNAs, including LARP7, CDKN1A, KLF2, TIA1, DDX3X, CDK, and QKI [94,95,96,97,98]. An analysis of the TCGA database showed that 1097 lncRNAs are expressed in BC, with 1510 differentially expressed lncRNAs in TNBC cells, 35 plasma lncRNAs in TNBC, and 672 in non-TNBC cells [14]. Some lncRNAs are directly linked to prognosis in TNBC, including FOXCUT, LINC00299, AP000924.1, AC091043.1, AL354793.1, AC010343.3, and FGF10-AS1 [14]. Plasma-specific lncRNAs are also used for diagnosis of TNBC, such as UCA1, ANRIL, and HIF1A-AS2 [30]. lncRNAs associated with lymph node metastasis, such as LINC000173, LINC00096, ZEB2-AS1, HIF1A-AS2, HULC, LUCAT1, SNHG12, MALAT1, HOTAIR, HIF1A-AS2, LINC00096, ADPGK-AS1, and ZEB2-AS1, have also shown importance in diagnosis and prognosis [11,14,30,49].

### 3.3. Importance of lncRNAs in Treatment

lncRNAs affect the response to treatments such as chemotherapy, immunotherapy, and radiotherapy [99]. H19 is expressed in patients with TNBC during neoadjuvant chemotherapy and is related to effective clinical outcomes. LINK-A expression is linked to response to pembrolizumab treatment in patients with TNBC because its decreased expression reduces CD8^+^ T-cell infiltration [59]. These lncRNAs act as biomarkers for treatment response in patients with TNBC. LncAFAP1-AS1 expression has been observed in patients with TNBC who received radiotherapy after surgery, and this lncRNA acts as biomarker for radiotherapy [82]. Moreover, lncRNAs are involved in angiogenesis. LINC01133 expression is induced by mesenchymal stem/stromal cells that adjoin TNBC cells [33]. lncRNAs are actively involved in the regulation of cell proliferation and apoptosis as well as drug resistance in TNBC [16,44,47,61,99]. DRHC and HOTAIR inhibit TNBC growth and development [31]. HOTAIR plays a role in the invasion and migration of TNBC cells and is used as a biomarker for TNBC metastasis in circulation and tissues, indicating poor survival and response [31,32]. DRHC inhibits TNBC cell proliferation by downregulating the expression of HOTAIR, whereas HOTAIR does not affect the expression level of DRHC. H19 expression is reduced in TNBC cells, whereas PTCSC3 expression is not altered by H19 overexpression [61]. HIST2H2BC and SNRPEP4 were identified in 165 frozen tissue samples by transcriptome microarrays; these lncRNAs are involved in taxane chemotherapy in patients with TNBC. Increased miR-377-3p expression delays TNBC progression by regulating the inc00339/miR-377-3p/HOXC6 axis and inhibits TNBC proliferation and apoptosis. Therefore, it is used as therapeutic target. HIF1A-AS2 expression is upregulated in TNBC mammary tissue, which is linked to overall survival. HOTAIR is closely associated with androgen receptor expression and used as a therapeutic strategy to prevent metastasis. The miR-199a/FOXP2 pathway is induced by LINC01133 and triggers the proliferation of TNBC cells. Various lncRNAs act as stem cell markers, such as DANCR, LINC01638, LINC-ZNF469-3, NEAT1, NRAD1, and ASRPS [75,87]. Some lncRNAs promote vasculogenic mimicry, providing growth supplementation for tumour formation in TNBC. TP73-AS1, which is activated by the miR-490-3p/TWIST1 pathway, is one example. LINK-A alters glycolysis by mediating HIF1α phosphorylation at Tyr565 and Ser7 [3,16,44,47]. MANCR inhibits DNA damage and prevents disease progression [66]. AWPPH is involved in the prevention of tumourigenesis upon treatment with carboplatin; AWPPH small interfering RNA (siRNA) silencing leads to increased chemosensitivity in TNBC [10,56]. TUG1 induces the expression of miR-197, reduces the activation of WNT signalling, and enhances TNBC cell sensitivity to cisplatin [75]. These findings demonstrate the importance of lncRNAs in the prevention of tumourigenesis. More studies are required to explore lncRNA treatment options. Early studies showed that HOTAIR recruits the polycomb repressive complex 2 to its target genes through the CoREST/REST H3K4 demethylase complex [75].

## 4. Nanoparticle-Based Targeted Therapy with ncRNAs for TNBC

**Figure 7:**
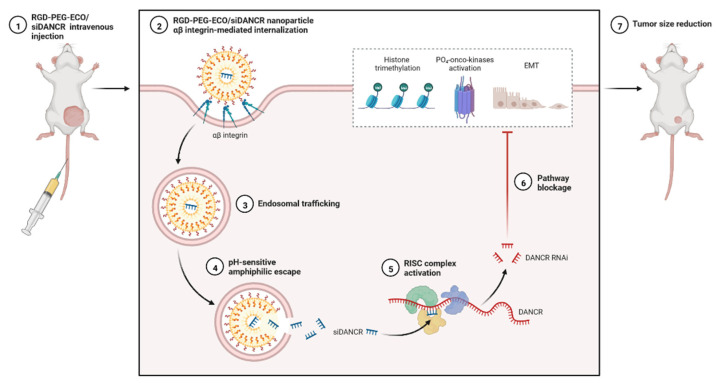
Expression analysis of RGD-PEG-ECO/siDANCR nanoparticles in an animal model of TNBC.

lncRNAs are versatile, able to exert multilevel gene regulation, and have emerged as therapeutic targets for clinically complicated TNBC cases (Figure 6) [25]. DANCR may potentially be used to reduce the limitations of monotherapy in TNBC networks and to lower the risk of side effects in healthy tissues [58]. DANCR is targeted by RNA interference (RNAi) (Figure 5 and Figure 6). The main challenge of RNAi therapy is targeted delivery; a non-viral siRNA-based delivery system has shown limited efficacy and temporary expression [94]. Amino acid-based lipid carriers have shown promising results in siRNA, nucleic acids, and CRISPR/Cas-based approaches [95]. DANCR overexpression was established in TNBC using RGD-PEG-ECO/siDANCR nanoparticles for effective cytosolic delivery of siDANCR [96]. Injection of the RGD-PEG-ECO/siDANCR nanoparticles led to reduce the progression of disease severity (Figure 7) [97]. In another study, LINC00511-siRNA was used to deliver siRNA in patients with TNBC [97]. Nanoengineered platforms were used to deliver lncAFAP1-AS1 siRNA (siAFAP1-AS1) to reverse radioresistance and increase the efficacy in TNBC tumour models [98]. These findings demonstrate that RNA nanoparticle-based targeted therapy can be more effective in TNBC. In conclusion, various lncRNAs are abnormally expressed and used in the treatment of TNBC, including ASOs, LNA, or RNA nanotechnology targeting lncRNAs.

## 5. LncRNAs Involved in The Regulation of Drug Resistance

**Figure 8:**
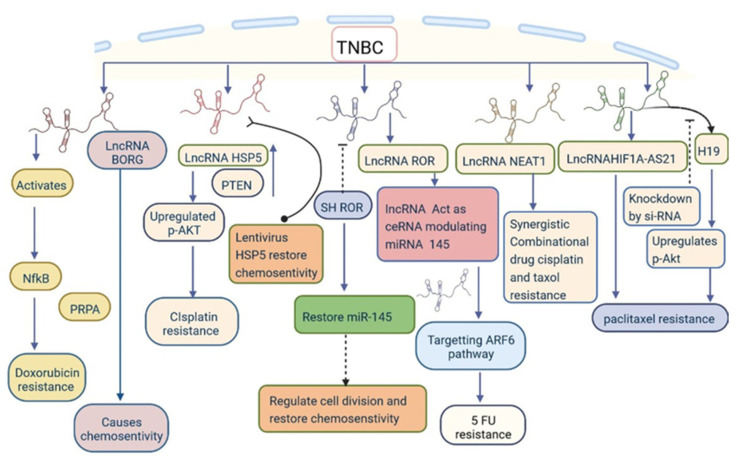
lncRNAs are involved in drug resistance through various mechanisms.

lncRNAs can alter the genetic regulation that may lead to the development of drug resistance. GAS5 promotes drug resistance to adriamycin, paclitaxel, and cisplatin [3,16,44,47]. Furthermore, GAS5 expression is reduced by mTORC1/mTORC2 (AZD8055) and PI3K/mTOR (BEZ235) inhibitors [99]. HOTAIR expression is controlled by EGFR/HER-2 inhibitor-based treatment such as lapatinib or the c-ABL inhibitor imatinib. Suppression of HOTAIR expression by the action of β-catenin on the HOTAIR promoter at the LEF1/TCF4-binding site increases drug resistance to combined therapy [100]. LINC01139 binding to the pleckstrin homology domain of AKT leads to hyperactivation and causes drug resistance to AKT inhibitors, which are commonly used for treatment of patients with TNBC [101]. Another treatment option for TNBC is immunotherapy-based treatments. LINK-A causes drug resistance by activating LINK-A–PKA–TRIM71 signalling, reducing the efficacy of immune checkpoint inhibitor-based treatments [102]. HIF1A-AS2 and AK12 4454 also cause drug resistance in TNBC cells [30]. H19 and NEAT1 induce resistance to paclitaxel through the AKT signalling pathway [16,61]. BORG causes resistance to doxorubicin through NF-κB signalling (Figure 8) [4,74]. DNA damage and repair is a complex process, and various signalling pathways are involved; impairment of this process leads to tumour development [103]. LINP1 participates in DNA double-strand break repair mechanisms using a scaffold linked up with Ku80 and DNA-PKcs, which prevents resistance to ionizing radiation [104]. INP1 prevents resistance to radiotherapy in TNBC [105]. The knockout of PCAT6 enhances the radiosensitivity of TNBC cells via the miR-185-5p/TPD52 axis [50]. These findings demonstrate the potential use of lncRNAs for the regulation of drug resistance in patients with TNBC (Table 2 and Figure 8). Further studies are required to investigate the roles of other lncRNAs in drug resistance.

## 6. Future Perspectives of lncRNAs as Potential Diagnosis and Pharmacological Tools/Targets

Different aspects of lncRNAs remain unclear, including their expression patterns in TNBC cells and their role in the modulation of mRNA coding genes [14,25]. Understanding their molecular heterogeneity would be helpful for developing novel drugs [113]. Single-stranded oligonucleotide antagonists targeting ASBEL have been designed to improve the half-life of the lncRNA in the serum. lncRNA expression is also regulated by tyrosine kinase receptors (TKRs) and non-TKRs through the simultaneous action of multiple genes; more studies are required to identify unknown mechanisms for the simultaneous targeting of multiple genes [114]. Moreover, studies focusing on molecular mechanisms are needed to improve our understanding of how FDA-approved chemotherapeutic agents for malignant neoplasms exert their regulatory action through epigenetic mechanisms on TNBC. The expression level of lncRNAs is dysregulated by chromosomes 1 and 10 via an unknown mechanism, which also needs to be explored [112]. The co-localization of lncRNAs plays a major role in TNBC progression and endocrine-based resistance therapy; we need to consider co-expressed lncRNAs to identify possible strategies for better diagnosis and treatment options [115]. Some lncRNAs are used as biomarkers; high-throughput analysis of lncRNAs by next generation sequencing in TNBC cells should be conducted using cell lines and animal models to identify abnormally expressed lncRNAs in TNBC. lncRNA replacement therapy could potentially be used to restore tumour-suppressive lncRNAs [115]. A CRISPR/Cas9 (Clustered Regularly Interspaced Short Palindromic Repeats/CRISPR-associated protein 9)-based approach can be used to reprogram transcription regulatory network and immune regulations of lncRNAs [116,117,118] The co-expression patterns of lncRNAs with transcription and translation need to be further explored to identify genetic heterogeneity pathophysiology mechanisms for early diagnosis, drug discovery, and understanding the treatment response and drug resistance mechanisms in patients with TNBC.

## 7. Conclusions

Clinical management of patients with TNBC is difficult owing to aggressive tumour behaviour and histological heterogeneity. The biological behaviour of TNBC cells, including genetic and epigenetic regulation, is not fully understood. We need to discover novel molecular biomarkers and therapeutic targets for better treatment efficacy. Personalized therapy for TNBC patients is required to reduce TNBC progression. lncRNAs may prove to be very useful, as they play important roles in TNBC development and response to follow-up treatment. Considering the challenges of in vivo experimental designs, lncRNAs continue to be promising as biomarkers and potential therapeutic targets. Most lncRNAs exhibit low sequence conservation, which may limit the scope of efficacy. For instance, most lncRNAs are not common in humans or mice; therefore, loss-of-function experiments in mice are not feasible and positive clinical outcomes using this approach have remained limited. Accordingly, with the significant advances in the genetic study of lncRNAs, novel methods should be explored for diagnosis, therapy, and prognosis, but the potential clinical applications of lncRNAs are significant. RNAi is the most widely applied and efficient technology for targeting lncRNAs. To use lncRNAs in therapeutic settings, however, different technologies must be investigated, and more study is required. Compared to esiRNAs (endonuclease-made siRNA), conventional siRNAs show more off-target effects. Short hairpin RNA (shRNA), which is produced inside of cells, is another type of RNAi. When compared to esiRNAs, shRNAs have a significantly greater off-target effects and produce a silent response that may be temporary or sustained. Plasmid vectors containing shRNA or siRNA are used to transfect tumour cells. Therefore, toxicity and off-target effects are other limitations of the lncRNA delivery system. To overcome the limitations of current diagnosis and treatment strategies, additional research is required for the use of lncRNAs as diagnostic biomarkers and therapeutic targets in TNBC.

## Figures and Tables

**Table 1:** Important lncRNAs associated with triple-negative breast cancer.

S. N.	lncRNAs	Regulation of Expression	Clinical Importance	Potential Targets	Reference
1	HOTAIR	Upregulation	Increase cell invasion and migration	LEF1/TCF4	[31]
2	DRHC	Downregulation	Reduce cell proliferation	HOTAIR	[32]
3	LINC01133	Upregulation	Promote phenotypic features like cell stem cells (CSCs)	KLF4	[33]
4	LINC01096	Upregulation	Encourage cell invasion	miR-3130-3p	[34]
5	HEIH	Upregulation	Increase cell proliferation and prevent cell death	miR-4458/SOCS1	[35]
6	ARNILA	Downregulation	Invasion and metastasis	miR-204/SOX4	[36]
7	LINC02095	Upregulation	Promote cell proliferation	SOX9	[37]
8	WT1-AS	Downregulation	Inhibit cell migration and invasion	TGF-β1	[38]
9	GAS5	Downregulation	Promote cell apoptosis	miR-378a-5p/SUFU	[39]
10	CCAT1	Upregulation	Encourage cell division	miR-218/ZFX	[40]
11	ASRPS	Downregulation	Inhibit angiogenesis and cell proliferation	STAT3	[41]
12	AND2-AS1	Downregulation	Inhibit angiogenesis inhibit cell division	RUNX2	[42]
13	POU3F3	Upregulation	Promote cell proliferation and inhibit cell apoptosis	Caspase-9	[43]
14	NEF	Downregulation	Inhibit cell migration and invasion	miR-155	[44]
15	ZEB2-AS1	Upregulation	Promote cell proliferation, metastasis, and EMT	ZEB2	[45]
16	LINC0009	Upregulation	Increase cell proliferation and invasion	miR-383-5p/RBM3	[46]
17	ANRIL	Upregulation	Increase cell proliferation and apoptosis	miR-448/KDM5B	[47]
18	SNHG12	Upregulation	Induce cell proliferation, migration, and apoptosis	MMP13	[48]
19	LUCAT1	Upregulation	Encourage cell division, movement, and invasion	miR-5702	[49]
20	PCAT6	Upregulation	Radiotherapy resistance	miR-185-5p/TPD52	[50]
22	HULC	Upregulation	Promote metastasis	MMP-2, MMP-9	[51]
23	PAPAS	Upregulation	Induce cell migration and invasion	miR-34a	[52]
24	HCP5	Upregulation	Increase cell proliferation; reduce cell apoptosis	miR-219a-5p/BIRC3	[53]
25	NRAD1	Upregulation	Stimulate cell proliferation and CSC-like phenotypic traits	miR-219a-5p/BIRC3	[54]
26	SNAR	Upregulation	Stimulate cell division		[55]
27	AWPPH	Upregulation	Activate cell proliferation	miR-21; FZD7	[56]
28	sONE	Downregulation	Prevent cell proliferation	TP53/c-Myc	[57]
29	DANCR	Upregulation	Promote cell proliferation and invasion	miR-216a-5p	[58]
30	LINK-A	Upregulation	Increase resistance to immunotherapy, AKT inhibitors, and glycolysis reprogramming	PI3K/GPCR	[59]
31	MIR503HG	Downregulation	Reduce cell migration and invasion	miR-103/OLFM4	[60]
32	NEAT1	Upregulation	Increase cell apoptosis		[61]
33	PTCSC3	Downregulation	Prevent cell proliferation	H19	[62]
34	NRON	Downregulation	Inhibit cell proliferation	snaR	[63]
35	TROJAN	Upregulation	Promote cell proliferation and invasion	ZMYND8	[64]
36	NAMPT-AS	Upregulation	Increase cell metastasis	miR-548b-3p/NAMPT	[14]
37	MANCR	Upregulation	Promote cell proliferation; inhibit DNA damage		[65]
38	RMST	Downregulation	Prevent cell proliferation		[66]
39	SK AI1BC	Upregulation	Increase cell migration and invasion	K AI1	[67]
40	ROR	Upregulation	Promote cell invasion and metastasis	miR-145/ARF6	[68]
41	AIRN	Downregulation	Inhibit cell migration and invasion	Wnt/β-catenin/mTOR/PI3K	[69]
42	LINC-ZNF469-3	Upregulation	Promote cell invasion	miR-574-5p/ZEB1	[70]
43	PDCD4-AS1	Downregulation	Inhibit cell proliferation and migration	PDCD4	[71]
44	HOST2	Downregulation	Inhibit cell proliferation	et-7 b/CDK6	[72]
45	BORG	Upregulation	Promote doxorubicin resistance	RPA1	[73]
46	PVT1	Upregulation	Promote cell proliferation and migration, and EMT	p21, KLF5/β-catenin	[24]
47	H19	Upregulation	Promote paclitaxel resistance and CSC-like phenotypic traits	Akt	[62]
48	TP73-AS1	Downregulation	Promote cell vasculogenic mimicry	miR-490-3p/TWIST1	[74]
49	TUG1	Downregulation	Enhance cisplatin sensitivity	miR-197/NLK	[75]
50	MIR100HG	Upregulation	Promote cell proliferation	p27	[76]
51	LINC01638	Upregulation	Promote cell proliferation	c-Myc	[77]

**Table 2:** lncRNAs participate in the drug resistance of TNBC treatment.

S.N.	lncRNA	Expression Patterns	Pathway/Target	Expression Pattern Drugs	Reference
1	H19	Upregulation	H19/Let-7/LIN28 axis	Anthracycline, paclitaxel, fulverstrant, doxorubicin tamoxifen	[19]
2	NEAT1	Upregulation	miR-211/HMGA2	Paclitaxel, 5-FU, cisplatin	[61]
3	GAS5	Downregulation	miR-21/mTOR/	Adriamycin, trastuzumab, tamoxifen, paclitaxel	[91]
4	LINK-A	Upregulation	PIP3/GPCR/cAMP/PKA/ TRIM71/PLC	Immune checkpoint blockers	[101]
5	UCA1	Upregulation	Wnt/b-catenin signalling	Trastuzumab, tamoxifen	[89]
6	LINP1	Upregulation	Caspase-9/Bax	Doxorubicin, 5-FU, tamoxifen	[105]
7	Linc-ROR	Upregulation	miR-194-3p/MECP2	Tamoxifen, paclitaxel, 5-FU	[106]
8	TMPO-AS1	Upregulation	ER	Endocrine therapy	[107]
9	DCST1-AS1	Upregulation	ANXA1	Doxorubicin, paclitaxel	[108]
10	TINCR	Upregulation	miR-125b/ERBB2	Trastuzumab	[109]
11	HOTAIR	Upregulation	ER	Tamoxifen, doxorubicin, trastuzumab,	[100]
12	AFAP1-AS1	Upregulation	AUF1/ERBB2	Trastuzumab	[98]
13	AGAP2-AS1	Upregulation	CBP/ MyD88/H3K27/NF-kB	Trastuzumab	[110]
14	AK124454	Upregulation		Paclitaxel	[111]
15	NONHSAT057282	Upregulation	ELF1 and E2F1	Anthracycline	[112]
16	NONHSAG023333	Upregulation	ELF1 and E2F1	Anthracycline	[112]

## Abbreviations

Long non-coding RNA: lncRNA; Triple-negative breast cancer: TNBC; Breast cancer: BC; Luminal A: LA; Hormone receptor positive: HR+; Oestrogen receptor-positive: ER+; Human epidermal growth factor receptor 2: HER2; Human epidermal growth factor receptor 2 positive: HER2+; Epidermal growth factor receptor: EGFR; Vascular endothelial growth factor: VEGFR; Master regulator of cell cycle entry and proliferative metabolism:- c-Myc (MYC); Androgen receptor: AR; Cyclin E: CCNE; Mouse double minute 2 homolog: MDM2; Phosphatidylinositol-4,5-bisphosphate 3-kinase catalytic subunit alpha: PIK3CA; Membrane-associated guanylate kinase WW and PDZ domain containing I: MAGI; Serine/threonine kinase 3: AKT3; Myosin IIIA: MYO3A; Parkin RBR E3 ubiquitin protein ligase: PRKN; Inositol polyphosphate-4-phosphatase type II B: INPP4B; Phosphatase and tensin homolog: PTEN; Cyclin-dependent kinase inhibitor 2A: CDKN2A; Breast cancer gene 1: BRCA1; Breast cancer gene 2: BRCA2; Tumour protein p53: TP53; RB transcriptional corepressor 1: RB1; Myeloid/lymphoid or mixed-lineage leukaemia 3: MLL3; Ribonucleic acid: RNA; Deoxyribonucleic acid: DNA; Non-coding RNA: ncRNA; microRNAs: miRNAs; Ribosomal ribonucleic acid: rRNA; Transfer RNAs: tRNAs; Small interfering RNAs: siRNA; Small nuclear RNAs: snRNAs; Extracellular RNAs: exRNAs; Circular RNAs: circRNAs; Non-coding repressor of NFAT: NRON; Hepatocellular carcinoma up-regulated EZH2-associated long non-coding RNA: HEIH; HLA complex P5: HCP5; one of the most upregulated lncRNA: LINC00096; Nuclear paraspeckle assembly transcript 1: NEAT1; Associated with poor prognosis of hepatocellular carcinoma: AWPPH; Lung cancer-associated transcript 1: LUCAT1; Heart and neural crest derivatives expressed transcript 2 antisense RNA 1: HAND2-AS1; POU domain, class 3, transcription factor: POU3F3; Metastasis-associated lung adenocarcinoma transcript 1: MALAT1; Antisense non-coding RNA in the INK4 locus: ANRIL; Protein kinase B: AKT; Mitogen-activated protein kinases: MAPKs; Competing endogenous RNAs: ceRNA; Homo sapiens OSTN antisense RNA 1: OSTN-AS1; Competing endogenous RNA: ceRNA; Chordin-like 1: CHRDL1; High affinity immunoglobulin gamma Fc receptor IA: FCGR1A; Radical S-adenosyl methionine domain-containing 2: RSAD2; HIF1A antisense RNA 2: HIF1A-AS2; HOX antisense intergenic RNA: HOTAIR; Down-regulated in human cancers: DRHC; Long intergenic non-protein coding RNA 1133: LINC01133; Long intergenic non-protein coding RNA 1096: LINC01096; Long non-coding RNA high expression in hepatocellular carcinoma: lncRNA HEIH; Androgen receptor negatively regulated lncRNA: ARNILA; Long intergenic non-protein coding RNA02095: LINC02095; Wilms tumour protein antisense RNA: WT1-AS; Growth arrest specific 5: GAS5; Colon cancer-associated transcript 1: CCAT1; A small regulatory peptide of STAT3: ASRPS; Radical S-adenosyl methionine domain-containing protein 2: AND2-AS1; RNA-induced silencing complex: RISC; Lysine-specific demethylase 5B: KDM5B; Histone demethylase 1B: JARID1B; Trimethylation of lysine 4 on the histone H3 protein subunit: H3K4me3; Monomethylation of lysine 4 on the histone H3 protein subunit: H3K4me1; retinoblastoma protein: pRB; caveolin 1: CAV1; Homeobox protein Hox-A5: HOXA5; Stratifin: SFN; methyl group: CH3; Ras homolog gene family, member A: RhoA; Cyclin-dependent kinase inhibitor 1B: p27; Mir-100-Let-7a-2-Mir-125b-1 cluster host gene: MIR100HG; Triplex-forming oligonucleotides: TFO; Triplex-targeting ability: TTA; Plasmacytoma variant translocation 1: PVT1; Krüppel-like factors: KLF5; BRCA1-associated protein-1: BAP1; Basic helix–loop–helix: bHLH; Next-generation sequencing: NGS; Epidermal growth factor receptor family: ErbB; Phosphatidylinositol-3-kinase: PI3K; Mammalian target of rapamycin: mTOR; POU domain, class 3, transcription factor 3: POU3F3; Negative regulatory factor: NEF; Zinc finger E-box-binding homeobox 2 antisense RNA1: ZEB2 – AS1; Small nucleolar RNA host gene 12: SNHG12; Prostate cancer-associated transcript 6: CAT6; Hepatocellular carcinoma up-regulated long non-coding RNA: HULC; Pap fimbrial major pilin protein: papA; lncRNA human histocompatibility leukocyte antigen (HLA), complex P5: HCP5; non-coding RNA in the aldehyde dehydrogenase 1A pathway: NRAD1; Small NF90 (ILF3)-associated RNA I: SNAR-I; DNA and RNA binding protein: SON; Differentiation antagonizing non-protein coding RNA: DANCR; Long intergenic non-coding RNA for kinase activation: LINK-A; MIR503 host gene: MIR503HG; Long non-coding RNA (lncRNA) nuclear enriched abundant transcript 1: NEAT1; Papillary thyroid carcinoma susceptibility candidate 3: PTCSC3; Nicotinamide phosphoribosyl transferase: NAMPT; Mitotically associated long non-coding RNA: MANCR; Rhabdomyosarcoma 2-associated transcript: RMST; Small conductance Ca2+-activated K+ (SK) ATP binding cassette subfamily A member 1: SK AI1BC; Receptor tyrosine kinase-like orphan receptor: ROR; Antisense of IGF2R non-protein coding RNA: AIRN; Long intergenic non-protein coding zinc finger protein 469: LINC ZNF469; Programmed cell death 4 antisense RNA 1: PDCD4-AS1; Human ovarian cancer-specific transcript 2: HOST2; BMP/OP-responsive gene: BORG; H19 imprinted maternally expressed transcript: H19; lncRNA P73 antisense RNA 1: TP73-AS1; Taurine up-regulated 1: TUG1; Mir-100-Let-7a-2-Mir-125b-1 cluster host gene: MIR100HG; Homo sapiens zinc finger MYND-type containing 8: ZMYND8; Zinc finger protein x-linked: ZFX; Human olfactomedin 4: OLFM4; Actin filament-associated protein 1: AFAP1; Epithelial–mesenchymal transition: EMT; Studied nitric oxide synthase 3: NOS3; Glycogen synthase kinase-3 β: GSK3β; Krueppel-like factor 5/β: KLF5/β; Wingless-related integration site: Wnt; Rhabdomyosarcoma 2 associated transcript: RMST; Spindle and kinetochore associated complex subunit 1: *SKA1;* LIM homeobox: Lhx; Kelch domain containing 1: KLHDC1; Hypoxia inducible factor 1 subunit alpha: HIF1A; HIF1A antisense RNA 2: HIF1A-AS2; Urothelial cancer-associated 1: UCA1; Area under curve: AUC; SOX2 overlapping transcript: SOX2OT; SRY-box transcription factor 2: SOX2; Ras association domain family member 1: RASSF1; Enhancer of Zeste 2 polycomb repressive complex 2 subunit: *EZH2;* Suppressor of cytokine signalling 1: SOCS1; Adenosine diphosphate-dependent glucokinase antisense RNA 1: ADPGK-AS1; Retinoid X receptor: RXR; Neural precursor cell expressed developmentally down-regulated protein 4-1: NEDD4-1; Phosphatase and tensin homolog: PTEN; Cyclin-dependent kinases: CDKs; Homeobox C cluster 6: HOXC6; SUFU negative regulator of hedgehog signalling: SUFU; La ribonucleoprotein 7- Transcriptional Regulator: LARP7; Cyclin Dependent Kinase inhibitor 1A: CDKN1A; T-cell intracellular antigen 1:TIA1; DEAD-box helicase 3 X-Linked: DDX3X; QKI, KH domain containing RNA binding: QKI; Fibroblast growth factor 10: FGF10; FGF10 antisense RNA 1: FGF10-AS1; Neoadjuvant chemotherapy: NAC; Mesenchymal stem/stromal cells: MSCs; Papillary thyroid carcinoma susceptibility candidate 3: PTCSC3; Human putative histone H2B type 2-C: HIST2H2BC; Small nuclear ribonucleoprotein polypeptide E pseudogene 4: SNRPEP4; Overall survival: OS; Androgen receptor: AR; Twist family BHLH transcription factor 1: TWIST1; Hypoxia-inducible factor 1-alpha: HIF1α; Mitotically associated long non-coding RNA: MANCR; Associated with poor prognosis of hepatocellular carcinoma: AWPPH; Nemo-like kinase: NLK; Polycomb repressive complex 2: PRC2; Repressor element-1 silencing transcription factor: REST; Co-element-1 silencing transcription factor: CoREST; RNA interference: RNAi; Clustered regularly interspaced short palindromic repeats: CRISPR; CRISPR-associated protein 9: Cas 9; Ras association domain family member 1: RASSF1; Terminal differentiation–induced non-coding RNA: TINCR; Urothelial cancer-associated 1: UCA1; Polycomb repressive complex 2: PRC2; Antisense oligonucleotides: ASOs; Locked nucleic acid: LNA; Growth-stasis-specific transcript 5: GAS 5; Pan-class I PI3K and mTOR kinase inhibitor (Dactolisib): BEZ235; Lymphoid enhancer binding factor 1: LEF1; Transcription factor 4: TCF4; Tripartite motif-containing 71: RIM71; DNA double-strand break: DSB; Tyrosine kinase receptors: TKRs.
